# Effects of Metabolic Disorders in Immune Cells and Synoviocytes on the Development of Rheumatoid Arthritis

**DOI:** 10.3390/metabo12070634

**Published:** 2022-07-11

**Authors:** Alexander V. Blagov, Andrey V. Grechko, Nikita G. Nikiforov, Alexander D. Zhuravlev, Nikolay K. Sadykhov, Alexander N. Orekhov

**Affiliations:** 1 Laboratory of Angiopathology, Institute of General Pathology and Pathophysiology, Russian Academy of Medical Sciences, 8 Baltiiskaya Street, 125315 Moscow, Russia; nikiforov.mipt@googlemail.com (N.G.N.); drawnman@mail.ru (N.K.S.); 2 Federal Research and Clinical Center of Intensive Care Medicine and Rehabilitology, 14–3 Solyanka Street, 109240 Moscow, Russia; avg-2007@yandex.ru; 3 Petrovsky National Research Centre of Surgery, AP Avtsyn Institute of Human Morphology, 117418 Moscow, Russia; zhuravel17@yandex.ru

**Keywords:** rheumatoid arthritis, autoimmune disease, inflammation

## Abstract

Rheumatoid arthritis (RA) is a progressive autoimmune disease that affects the joints. It has been proven that, with the development of RA, there are changes in the metabolism of cells located in the focus of inflammation. In this article, we describe the connection between metabolism and inflammation in the context of rheumatoid arthritis. We consider in detail the changes in metabolic processes and their subsequent immunomodulatory effects. In particular, we consider how changes in mitochondrial functioning lead to the modulation of metabolism in rheumatoid arthritis. We also describe the main features of the metabolism in cells present in the synovial membrane during inflammation, and we discuss possible targets for the therapy of rheumatoid arthritis.

## 1. Introduction

Rheumatoid arthritis (RA) is a chronic autoimmune disease that leads to the development of a vigorous inflammatory reaction in the synovial membranes of the joints, which ultimately leads to the destruction of the joints [1]. The aetiology of RA is complex and most likely associated with the interaction of genetic predisposition and environmental factors, such as infectious agents, smoking, being overweight, etc. [1]. The most-studied genetic factor in the development of RA is the HLA-DRB1 gene, which encodes the beta chain of the major histocompatibility complex (MHC) of Class II, and it is involved in the recognition of native and foreign proteins [1]. The incidence of RA in the world is 1%, which characterizes it as one of the most common autoimmune diseases [1]. RA leads to a high level of disability in patients, associated not only with damage to the articular structures, but also with the development of systemic inflammation (in 40% of patients with RA), which affects other organs, including the skin, eyes, blood vessels, heart, and lungs, amongst others [2]. Patients with RA have an increased risk of developing osteoporosis, cardiovascular disease, and lymphoma [2].

**Figure 1:**
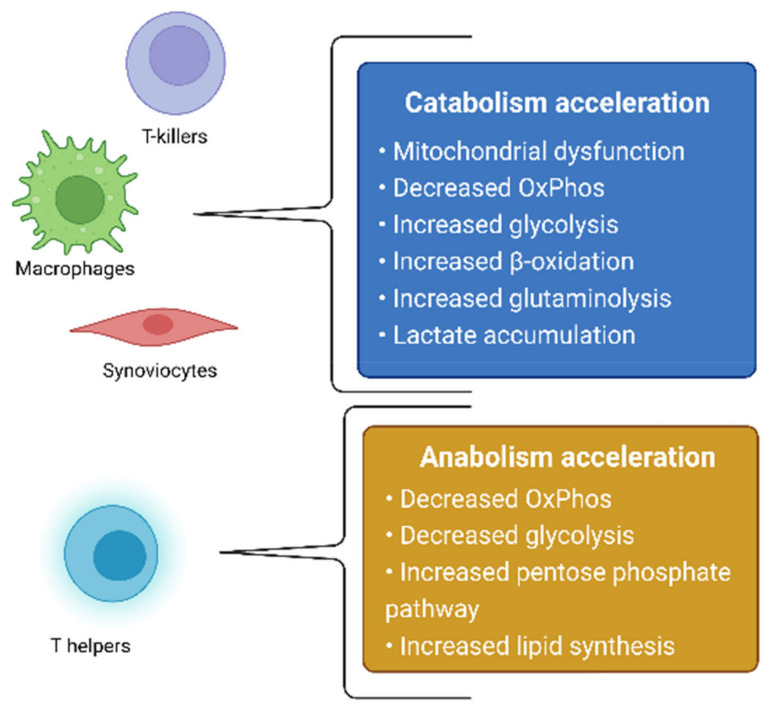
Summarized metabolic changes for cells involved in the pathogenesis of RA.

The treatment of RA is aimed at either the systemic suppression of inflammation via the use of glucocorticosteroids, nonsteroidal anti-inflammatory drugs, cytostatics that block white-blood-cell division, or at the targeted inhibition of molecular inflammatory targets—TNF-α, IL-6, IL-12, and JAK—with the use of monoclonal antibodies and JAK kinase inhibitors [3,4,5]. RA itself, as well as the therapeutic agents used for its treatment, causes changes in the state of the immune system, which can lead to an increased vulnerability of the body to infectious diseases [2], thus further worsening the patient’s well-being. Based on this, the development of new drugs, which neither have pronounced immunosuppressive properties nor affect other branches of the pathogenesis of RA, remains relevant. One of the possible therapeutic effects in the treatment of RA is the effect on the metabolic activity of cells involved in the pathogenesis of RA. The metabolism of cells in the focus of inflammation changes in RA, which is directly related to the development of mitochondrial dysfunction [6]. In this review, we will consider changes in metabolic processes in RA, as well as in cellular models based on this hypothesis. In addition, possible therapeutic strategies aimed at cellular metabolites will be proposed. Summarized metabolic changes for cells involved in the pathogenesis of RA are presented in Figure 1.

## 2. Metabolic Disorders in Rheumatoid Arthritis Connected with Mitochondrial Dysfunction

Mitochondria are important cellular organelles, providing energy to all biological processes occurring in the cell. The process of energy exchange in the cell is called cellular respiration, and it includes three main stages: glycolysis, the Krebs cycle, and oxidative phosphorylation. Glycolysis is the only one of these processes that does not occur in the mitochondria. During glycolysis, two pyruvic acid molecules are formed from one glucose molecule, which later take part in the Krebs cycle, and energy is stored in the form of two ATP molecules [7]. During the Krebs cycle, which is carried out in the mitochondrial matrix under the action of mitochondrial enzymes, a number of chemical reactions of intermediate metabolites takes place, as a result of which, the recovery of the byproducts flavin adenine dinucleotideFADH2 and nicotinamide adenine dinucleotide (NADH) occurs [7]. These byproducts participate in oxidative phosphorylation, which is carried out by transferring electrons between the electron transport chain carrier proteins (ETC) to molecular oxygen on the inner mitochondrial membrane. Electron transfer activates the transfer of protons from the mitochondrial matrix to the intermembrane space, which increases the electrochemical gradient and promotes the generation of energy, which is released in the form of ATP during reverse proton transport to the matrix via ATP synthase [8].

Despite the fact that most of the mitochondrial proteins are encoded by nuclear DNA, some subunits of enzymatic ETC carriers are encoded in the mitochondrial genome [7]. Due to the lack of DNA repairability and the production of reactive oxygen species (ROS) as a byproduct of oxidative phosphorylation, mitochondrial DNA is highly sensitive to mutations [7]. Mutations of genes encoding proteins involved in cellular respiration are the cause of a number of diseases associated with impaired energy metabolism in cells [9]. The pathogenesis of rheumatoid arthritis is also associated with various metabolic changes occurring in the cells present in the focus of inflammation. Next, we will discuss in detail each process of cellular respiration along with its disorders in rheumatoid arthritis.

### 2.1. Hypoxia

Oxygen is the main oxidizing agent in cellular respiration reactions. Under conditions of oxygen starvation, which is called hypoxia, there is a disruption of oxidative phosphorylation and, as a result, a decrease in ATP production [10]. When inflammation occurs, the blood vessel structure of the synovial membrane is disturbed: due to the increased extravasation of white blood cells, endothelial cells peel off, which leads to a disruption of conductivity and, as a result, to a decrease in the delivery of oxygen and nutrients to the synoviocytes [10]. The increased influx of a large number of immune cells into the synovial membrane causes an increase in oxygen consumption, which leads to even greater hypoxia.

Hypoxia also leads to an increase in the production of ROS that damage mitochondria, which leads to the release of the DAMP (damage-associated molecular pattern) molecules that can cause non-infectious inflammation [11]. Additionally, DAMPs are released when cells are directly destroyed. The main mitochondrial DAMPs are ATP, mitochondrial DNA, and N-formyl peptide [12]. Released from the mitochondria, they are able to activate innate immune receptors, which leads to the secretion of the pro-inflammatory cytokines IL-1β and IL-18, both of which are involved in autoimmune inflammation in RA [10].

Damage to mitochondrial DNA caused by ROS can also lead to mutations in the mitochondrial genome, which results in the disruption of cellular respiration. Increased DNA damage and the accumulation of dysfunctional mitochondria in white blood cells and synoviocytes have been observed in several studies in patients with RA [13]. An increased frequency of mitochondrial DNA mutations in the MT-ND1 gene encoding the NADH dehydrogenase subunit, the largest ETC complex, has been observed in patients with RA [14]. Anti-inflammatory drug therapy has also been shown to reduce hypoxia [15].

### 2.2. Glycolysis

Hypoxia leads to changes in the metabolism of immune cells. Because of hypoxia, energy metabolism occurs mainly by glycolysis, without affecting oxidative phosphorylation [10]. This activates the transcription factor HIF-1α, which increases the expression of glucose transporters and glycolytic enzymes [10]. Thus, there is an increase in the expression of the glucose transporter GLUT1 in the CD4+ T-lymphocytes and B-lymphocytes in patients with RA [6], and the T-helper cells, rendered defective by GLUT1, have a reduced proliferation [16]. At the same time, GLUT1 negative CD8 + T-lymphocytes show a similar cytotoxic effect as that of the wildtype, which suggests compensation for glucose uptake due to the work of other carriers: GLUT3 and GLUT6 [16].

Due to the action of HIF, the concentration of lactate in the cytoplasm increases, which lowers the pH medium. The occurrence of acidosis leads to an increase in the mutation frequency, which can also cause mitochondrial dysfunction [10]. It has also been shown that the increased lactate production by monocytes and macrophages leads to the increased expression of the proinflammatory cytokines, IL-6 and IL-23, which activate the proliferation of Th17 lymphocytes, which are one of the main populations of immune cells in RA [17]. In addition, lactate inhibits the migration of CD4+ and CD8+ T-lymphocytes, thus contributing to the retention of T-lymphocytes in the focus of inflammation [18]. An increased level of activity of the enzyme lactate dehydrogenase A (LDH), which activates the conversion of pyruvate to lactate, has also been detected in the serum and synovial fluid of patients with RA [19]. LDH promotes the maturation of T cells along the Th1 pathway and increases the production of IFN-γ via epigenetic action [20].

Despite the fact that glycolysis is a much less productive process, in terms of the amount of ATP produced, as compared to oxidative phosphorylation, it neither requires oxygen nor the participation of mitochondria, and it occurs much faster. In conditions of hypoxia, an increase in the number of dysfunctional mitochondria, and the increased proliferation of immune cells, it is the preferred way to obtain energy [21].

### 2.3. The Krebs Cycle

In addition to the production of electron transport mediators NADH and FADN2, which are involved in the functioning of ETC, intermediate metabolites are created during the Krebs cycle, which are then used for processes occurring in the cell, nucleus, and extracellular space [22]. One of the metabolites of the Krebs cycle is succinate, which is formed from succinyl-CoA and then converted to fumarate by the enzyme succinate dehydrogenase [6]. It is known that succinate can accumulate and be produced by macrophages in an increased concentration as a result of a disruption of the Krebs cycle due to the inhibition of the enzyme succinate dehydrogenase, which increases the inflammatory response [22]. At the same time, one of the important steps in the development of inflammation in RA is the binding of the succinate molecule to the GPR91 receptor, which is expressed in various types of immune cells [22]. Thus, it has been shown that, in GPR91 mice, neutrophil infiltration and the concentration of proinflammatory cytokines in the joint both decreased, as did Th17 cells [23]. Playing the role of an inflammatory agent in innate immunity, succinate seems to act in the opposite manner on adaptive immunity [22]. In T cells in RA, there is also a disruption of the Krebs cycle, but it occurs because of the SUCLG2 gene repression encoding the GTP-specific beta-subunit of succinyl-CoA synthetase, and as a result, the accumulation of α-ketoglutarate, acetyl-CoA and citrate occurs in T-cells [6]. Citrate also accumulates in macrophages due to the suppression of the enzyme isocitrate dehydrogenase activity [24]. The accumulation of these metabolites in T cells increases the acetylation of tubulin and leads to increased migration of T cells into the synovial membrane [25]. Thus, disruptions of the Krebs cycle that occur in RA, different for different cell types, eventually lead to the same outcome—an increase in the inflammatory response. In RA patients, decreases in the concentrations of various Krebs cycle enzymes were found, which are associated with a general decrease in Krebs cycle activity in RA [26].

### 2.4. Oxidative Phosphorylation

Under conditions of hypoxia in RA, there is a decrease in mitochondrial activity through the activation of transcription factor HIF-1α, which is aimed at reducing the excess production of ROS in Complexes I and III of ETC [27]. At the same time, the activity of oxidative phosphorylation decreases, which is associated with a decrease in the expression of the subunits of the ETC complexes [27]. In addition to the epigenetic inhibition of ETC activity in RA, the direct inhibition of ETC complex enzymes involving NO has also been observed. Thus, NO is able to reversibly inhibit the activity of cytochrome c oxidase as a result of competition for the binding site with oxygen [28]. NO also irreversibly inhibits the work of Complexes I and II, directly modifying cysteine residues during nitrosylation [28]. Signal pathways involving NO are also associated with a decrease in the number of Complex I subunits [29]. In the lymphocytes of patients with RA, an increased expression of induced NO synthase and NO production was observed, which suggests a possible role of NO in inflammation in RA [30,31,32].

Mutations of enzyme subunits involved in the process of oxidative phosphorylation are an additional reason for the disruption of the ETC [14,33]. As a result, electron leakage occurs during transmission through the ETC, which leads to the generation of ROS and the activation of the NLRP3 inflammasome, which activates the production of pro-inflammatory cytokines IL-1β and IL-18, both of which are directly involved in the inflammatory response in RA [6]. In addition to activating the innate branch of immunity, mitochondrial mutations can directly lead to the activation of adaptive immunity. Mitochondrial peptides with altered amino acid sequences due to mutations can act as autoantigens and trigger adaptive immunity through presentation to T-lymphocytes as part of MHC molecules, which, in addition to activating immune cells, leads to the formation of autoantibodies [34,35]. It has been shown that the frequency of mitochondrial mutations is significantly increased in patients with rheumatoid and psoriatic arthritis in comparison with healthy people [14,36]. Also, the frequency of mitochondrial mutations has been positively associated with the levels of TNFα and IFN-γ in the synovial fluid. In addition to the new, mutated variants that result from DNA damage during hypoxia and inflammation, there are certain mitochondrial haplotypes associated with the development of RA. They are mainly represented by genes encoding the components of the ETC [37,38]. Thus, in RA, a cyclical relationship is observed, in which inflammatory reaction leads to a decrease in the effectiveness of oxidative phosphorylation, which causes a new increase in inflammation.

### 2.5. Fatty Acid β-Oxidation

In addition to glucose, fatty acids can act as a substrate for energy production [6]. In the process of fatty acid β-oxidation, which occurs in the mitochondrial matrix, acetyl-CoA is formed, which enters the Krebs cycle, and NADH and FADN2 are released, which directly enter the ETC reactions [6]. Fatty acids are an important source of energy for different types of immune cells [6]. It is known that the process of fatty acid oxidation changes in RA, but the exact mechanism of its disruption is not fully clear [6]. An important step that precedes direct oxidation is the entry of fatty acids into the mitochondria, which is carried out with the help of carnitine [21]. There is a suggestion that carnitine may play an immunomodulatory role in RA. Thus, in a study [39], when exogenous carnitine was exposed to human monocyte cell culture, there was an increase in the LPS-induced production of CCL20, the inflammatory chemokine with a strong chemoattractant effect on lymphocytes. Another study also found that carnitine levels were elevated in the synovial fluid of patients with RA [26]. However, the same study showed a decrease in the expression of enzymes involved in fatty acid oxidation, which may be due to the fact that the change in fatty acid metabolism differs for different cell types in RA. Thus, it is known that CD4+ T-lymphocytes in RA are characterized by the predominance of fatty acid synthesis over their oxidation [22]. This metabolic shift contributes to an increase in the migration of T-lymphocytes to the focus of inflammation [22,40].

### 2.6. Glutaminolysis

An additional source of energy can be the amino acid glutamine [41]. It enters the Krebs cycle through conversion to glutamate, and then to α-ketoglutarate through the process of glutaminolysis [42]. Glutamate affects the differentiation of T-lymphocytes and the polarization of macrophages, and it promotes the proliferation of synovial fibroblasts [6]. The mitochondrial enzyme glutaminase 1 (GLS1) catalyses the initial stage of glutaminolysis: the formation of glutamate from glutamine [6]. In a study [43], an increased expression of GLS1 was shown in patients with RA. In addition, knockdown of the GLS1 gene or pharmacological inhibition of the enzyme itself led to a decrease in synoviocyte proliferation, which facilitated the course of the disease in an experimental mouse model. Thus, glutaminolysis is generally increased in RA. Due to the increase in the number of immune and stromal cells in the focus of inflammation in conditions of low glucose content, glutamine can become the main source of energy [6]. Glutamine is especially important for the population of Th17 cells, which are one of the main immune cells responsible for the development of RA [44]. It has been shown that the level of glutamine uptake by T cells increases between 5- and 10-fold in RA [22]. The transport proteins SLC1A5, SLC38A1, and SLC38A2 are responsible for the uptake of glutamine by cells [22]. For SLC1A5 proteins, an immunomodulatory role has also been shown: they are involved in the differentiation of naive CD4+ T-lymphocytes into Th1 and Th17 types [45,46].

## 3. Models of Rheumatoid Arthritis Pathogenesis Based on Metabolic Changes in Different Cell Types

### 3.1. Synoviocytes

Under conditions of increased hypoxia in the synovial membrane, catabolism shifts from oxidative phosphorylation to glycolysis and glutaminolysis (in the case of glucose deficiency) in synoviocytes [47]. A study [48] showed a reduced level of cellular respiration in the synoviocytes of patients with RA, which was associated with mitochondrial depolarization and abnormal crista morphology. Mitochondrial dysfunction is an additional cause of the shift in energy metabolism from oxidative phosphorylation to glycolysis. It should be noted that glycolysis contributes to the accumulation of lactate, which acidifies the environment, which leads to an increase in the number of mitochondrial mutations and, accordingly, to an increase in the number of dysfunctional mitochondria [10]. In addition, the development of mitochondrial dysfunction in synoviocytes can be caused by the effect of pro-inflammatory cytokines released by immune cells in the synovial sac. A particularly strong effect has been shown with the participation of IL-17 released by Th-17: IL-17 inhibited the expression of the components of Complexes I and III of ETC and ATPase, which led to a complete disruption of the processes of oxidative phosphorylation [48]. Additionally, IL-17 induced the inhibition of apoptosis, which is considered as contributing to the formation of an invasive pannus characteristic of patients with RA [48]. In addition, a strong role in the appearance of dysfunctional mitochondria in synoviocytes belongs to nitric oxide, whose effect on ETC enzymes was described in detail above. In a study [49], the induced exposure of IL-1β and the oligomycin inhibitor to healthy synoviocytes resulted in the increased expression of proinflammatory mediators IL-8, PGE-2, and COX-2. This may be due to increased ROS generation caused by mitochondrial dysfunction, which leads to mitochondrial damage, the release of DAMPS, and the triggering of an internal inflammatory response. Thus, the inflammatory environment that occurs in the synovial membrane in RA initiates a change in metabolism and the disruption of mitochondrial functions in synoviocytes, which ultimately leads to the launch of an inflammatory reaction in the synoviocytes themselves and to an overall increase in inflammation.

### 3.2. Macrophages

In an inflamed joint with RA, macrophages are the most numerous cells [50]. Since macrophages work exclusively as effector immune cells and have final differentiation, they do not require increased division and biosynthesis, but they need increased energy consumption [50]. As a result, the metabolism of macrophages is shifted in the direction of energy metabolism. Peripheral blood macrophages in RA have an increased rate of both glycolysis and oxidative phosphorylation, as well as increased oxygen consumption [51]. At the same time, upon entering an inflamed joint in conditions of reduced oxygen content, macrophages begin to receive energy mainly by glycolysis with limited oxidative phosphorylation [6]. A great contribution to this is made by the factor HIF-1a, which is activated during hypoxia, and it promotes the transcription of glycolytic enzymes [10]. With a decrease in the glucose content in the focus of inflammation, which may be caused by a deterioration in the conductivity of blood vessels and an increase in competition for glucose among migrated immune cells, macrophages can use glutamine, and possibly fatty acids, as an energy substrate. the acidification of the macrophage cell environment with lactate contributes to the modification of the glycolytic enzyme pyruvate kinase, as a result of which, this enzyme penetrates into the nucleus and activates the STAT3 gene, which ultimately leads to the production of the pro-inflammatory cytokines IL-1β and IL-6 by macrophages, which play an important role in the development of inflammation in RA [50]. Additionally, there is an increase in the expression of the cathepsin K protease in macrophages, which is one of the factors in joint destruction in RA [51,52].

### 3.3. CD8+ T-Lymphocytes

CD8+ T-lymphocytes make an important contribution to the development of RA. Both for CD8+ T-lymphocytes of peripheral blood and for a similar population of cells located in the affected joint, an increase in glycolysis and lactate formation has been shown to correlate with a simultaneous decrease in oxidative phosphorylation [53]. At the same time, the cytotoxic lymphocytes of patients with RA showed a decrease in the potential of the mitochondrial membrane and a decrease in oxygen uptake, as well as an increase in the expression of glycolytic enzymes [53]. With a lack of glucose, CD8+ T cells are able to switch to glutaminolysis, which also ends in the formation of lactate [53]. This metabolic profile under conditions of hypoxia promotes the proliferation of CD8+ T-lymphocytes and their production of pro-inflammatory cytokines, as well as an increase in the expression of granzyme B. In addition, it has been described that cytotoxic T-lymphocytes are able to activate B-lymphocytes in the synovial membrane in patients with RA [53]. The glycolytic enzyme lactate dehydrogenase (LDH) has a particularly important effect on the functioning of CD8+ T-lymphocytes. Thus, it has been shown that the inhibition of LDH reduces the proliferation of CD8+ T-lymphocytes and their ability to activate B cells [53].

### 3.4. CD4+ T-Lymphocytes

**Figure 2:**
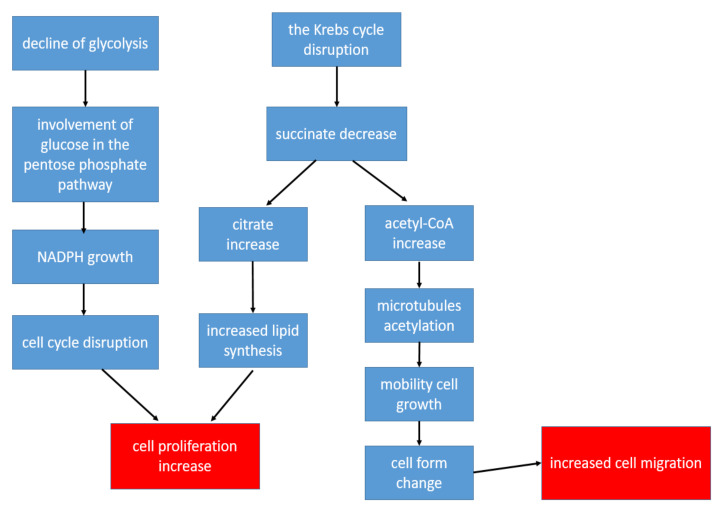
The key changes in the metabolism of T-helper cells leading to increased inflammation.

CD4+ T-lymphocytes are the main immune regulators in the development of inflammation in the affected joints in patients with RA. At the same time, the metabolism of T-helper cells is completely different from the metabolism of other populations of immune and stromal cells in RA: The metabolism of CD4+ T-lymphocytes is shifted in the direction of plastic metabolism, taking into account the need for increased biosynthesis for accelerated cell proliferation [22]. Therefore, in CD4+ T cells, there is a decrease in ATP production with the suppression of both glycolysis and oxidative phosphorylation, while glutaminolysis becomes the main way of obtaining energy [22]. Glucose, instead of glycolysis, is involved in the pentose phosphate pathway, which leads to the accumulation of NADPH, resulting in a change in cell-cycle control. This allows T-lymphocytes to enter into unlimited proliferation [54]. An important point is also the disruption of the Krebs cycle reactions, as a result of which, there is a decrease in the concentrations of some metabolites and an increase in the concentrations of others. Thus, the formation of succinate is disrupted, and the production of citrate and acetyl-CoA increases [25]. Citrate promotes the activation of lipid synthesis, which is necessary for the formation of cell membranes, which is an important factor in conditions of increased cell division [22]. Acetyl-CoA promotes the acetylation of cytoplasmic proteins. During the acetylation of tubulins (microtubule proteins), the structure of the cytoskeleton and the shapes of the lymphocyte change from rounded to elongated with the formation of uropods. As a result, T-helpers become hypermobile, which contributes to their increased extravasation from the peripheral blood to the area of inflammation [25]. The key changes in the metabolism of T-helper cells leading to increased inflammation are shown in Figure 2. Thus, despite a metabolic description that is very different from other cells, changes in the metabolism in CD4+ T-lymphocytes also contribute to the chronification of inflammation.

## 4. Therapy of Rheumatoid Arthritis Aimed at Regulating Metabolic Pathways

**Figure 3:**
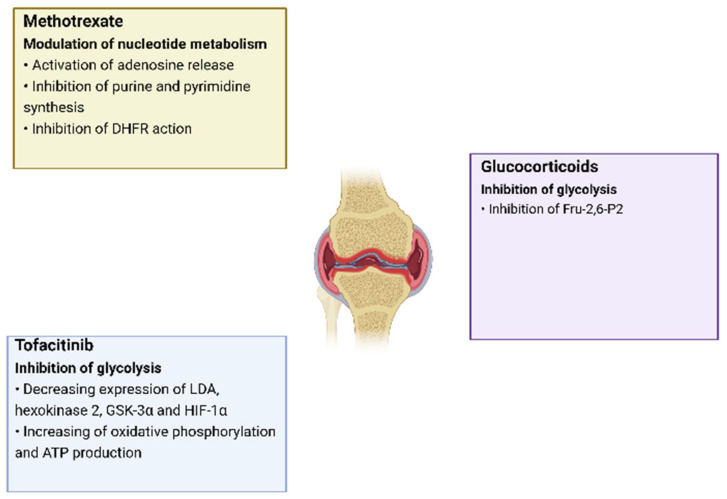
The effects of RA drugs on cell metabolism.

Changes in cell metabolism play an important role in the development of RA, as it leads not only to changes in the energy and plastic needs of cells, but also has an immunomodulatory function which, as a result, increases inflammation. Moreover, this pattern is characteristic of all groups of cells located in the inflamed synovial membrane. In addition, metabolic changes can occur in different ways, in which different metabolites and enzymes take part, which creates many options for the impact of targeted therapy in the treatment of RA. One of the proofs of the emerging metabolic disorders influence on the development of RA is the use of some already-registered drugs against RA, for which a recovering effect on metabolic disorders has been noted. Thus, tofacitinib directly affects mitochondrial and glycolytic genes, causing an increase in oxidative phosphorylation, an increase in ATP synthesis, and a decrease in glycolysis [55,56,57]. The mechanisms of effect on the metabolism by already-registered RA drugs are presented in more detail in Figure 3 [58].

The development of new drugs that have a modulating effect on cell metabolism can be an effective step in the fight against RA. At the same time, it should be understood that the effect on glucose or glutamine will not bring the desired effect since these substances are used as substrates for all types of cells of the human body [59]. At the same time, the effect on intermediate metabolic products, such as lactate, succinate, and citrate; the enzymes associated with their transformation; and their carrier proteins can become an effective immunomodulatory effect [22]. Thus, in a study on a mouse model, it was shown that dimethylmalonate (DMM) and 3-nitropropionic acid reduced the activity of succinate dehydrogenase, an enzyme involved in the oxidation of succinate [60]. Some possible variants of metabolic participants that can be selected as therapeutic targets are presented in Table 1. An additional opportunity for RA therapy can be provided by the use of ROS inhibitors and molecules that affect the expression of mitochondrial genes, coding subunits of ETC enzymes. Since autoimmune CD4+ T-lymphocytes are the main regulators of the inflammatory response in RA with a different metabolic shift from other cells in the synovial sac, the effect on specific metabolic targets characteristic of T-helpers can be a saving solution for slowing down or even stopping inflammation in the early stages of RA. Another possible therapeutic effect is the effect on the HIF-1α pathway associated with the development of hypoxia. Since the hypoxic environment affects all types of cells present in the focus of inflammation, the inhibition of this pathway can have a complex anti-inflammatory effect. An important addition to drug therapy in patients with RA may be the use of a specialized diet. Thus, in a clinical study [61], it was found that in patients with RA treated with antirheumatic drugs, the addition of a dairy-free, lactose-free, and gluten-free diet for 3 months led to decreases in CRP concentrations, the level of circulating leukocytes, and neutrophils in patients with RA, as well as a reduction of pain sensation.

## 5. Discussion

Despite the fact that RA is not a “classic” metabolic disease, the pathogenetic effect of changes in metabolism has been thoroughly proven. Like other chronic inflammatory diseases, one of the important targets in the pathogenesis of RA is the mitochondria. In the current review, the mitochondria are considered mostly as the main link in cellular metabolism. Indeed, a disruption of the cellular respiration processes occurring in the mitochondria—the Krebs cycle and oxidative phosphorylation—was observed in all types of cells in the synovial membrane of the inflamed joint. However, changes in metabolism are associated not only with mitochondria, but cover a wider range of processes, including glycolysis and anabolic reactions, as noted in this review. The cellular models of metabolic disorders considered here, as well as the descriptions of the metabolic processes themselves in the pathogenesis of RA, give a comprehensive picture for understanding the role of cellular metabolism in the development of RA. At the same time, some particular questions remain unclear, the answers to which can be encountered with a more thorough study of the topic of this review. First, it is worth studying in more detail the metabolic changes in Th17 T-helpers, which have increased activity in RA and other autoimmune processes. Second, an important step is to identify new, prevalent mutations associated with the genes encoding enzymes of the Krebs cycle and oxidative phosphorylation. Third, it is important to identify new cellular compounds that cause the epigenetic or chemical inhibition of cellular respiration reactions, which lead to a state of mitochondrial dysfunction. Fourth, it will be useful to compare the metabolic changes in cells, immune reactions in the focus of inflammation, and clinical manifestations of RA on the time-scale of the disease. An example of such a study is a study showing the relation between urinary metabolite profiles and inflammation levels in RA as measured by C reactive protein (CRP) detection [62]. The greatest correlation with inflammation has been shown for such metabolites as glucose, amino acids, lactic acid, and citric acid. The obtained results indicate the following changes in metabolism for patients with RA: accelerated glycolysis, disruption of the citric acid cycle, high activity of the urea cycle, and increased protein catabolism. The discovery of identified correlations may also be an essential prerequisite for the finding of new, effective RA markers. Fifth, an important broad area of research is the detailed study of the relationship between metabolic changes and the development of inflammation for other chronic inflammatory diseases. In addition, it is of great importance whether drugs used to treat RA can have a negative effect on metabolism, which, in turn, may be a prerequisite for the development of other diseases associated with metabolic disorders. Thus, in a clinical study [63], it was found that treatment of patients with RA with a combination of adalimumab and methotrexate or methotrexate alone led to a significant increase in cholesterol levels. It is also important to study the changes in the microbiome in RA and its treatment since metabolism also depends on the qualitative and quantitative composition of intestinal bacteria. Changing the microbiome in RA and its treatment have been demonstrated in a study [64]. In addition, repurposing drugs that modulate metabolism in other diseases may be an interesting therapeutic strategy. For example, fenofibrate, which activates fatty acid β-oxidation via PPARα activation and is used to treat cardiovascular disease, has been shown to protect against cartilage degradation and reduce inflammation in osteoarthritis as a drug with autophagic and senolytic activity [65].

## 6. Conclusions

Hypoxia caused by morphological changes in blood vessels and the influx of a large number of white blood cells are the initiating factors of metabolic changes in the cells present in the synovial membrane. Hypoxia, through various mechanisms, leads to the formation of dysfunctional mitochondria, as a result of which the Krebs cycle and oxidative phosphorylation are disrupted. An additional factor in the transition of most cells to glycolysis, in addition to mitochondrial dysfunction, is the formation of a hypoxic environment. In conditions of a decrease in the concentration of glucose, cells begin to use glutamine as the main source of energy. A special position is occupied by T-helpers, whose metabolism is shifted in the direction of anabolism due to the need for active biosynthesis and proliferation. Despite the different mechanisms of change in the metabolic pathways in cells caused by the initiation of an inflammatory reaction, the metabolic disorders themselves lead to increased inflammation, thus creating a cyclic connection leading to a setting of chronic inflammation. Metabolic mediators associated with these disorders may become new drug targets in the treatment of RA.

## Figures and Tables

**Table 1:** Some metabolic agents and their roles in the development of inflammation.

Metabolic Agent	Role in Inflammation
Lactate	growth of mitochondrial mutations, release of pro-inflammatory cytokines
Succinate	activation of innate immunity through binding to GPR91
Citrate	activation of the synthesis of lipids to create the membranes of new T-helper cells
Acetyl-CoA	increased migration of T cells to the synovial membrane
LDHA	activation of Th1 cell maturation, increase in IFN-γ production
(HIF) -1α	development of hypoxia in the cells of an inflamed joint
NO-synthase	synthesis of NO, which causes mitochondrial dysfunction
Carnitine	increased production of CCL20, which attracts lymphocytes
GLS1	important for the proliferation of synoviocytes and Th17 lymphocytes
SLC1A5	participation in the differentiation of naive CD4+ T-lymphocytes into Th1 and Th17 lymphocytes
Pyruvate kinase	production of proinflammatory cytokines IL-1β and IL-6 by macrophages
